# Pure-blue single-layer organic light-emitting diodes based on trap-free hyperfluorescence

**DOI:** 10.1038/s41563-025-02294-8

**Published:** 2025-07-21

**Authors:** Oskar Sachnik, Naomi Kinaret, Rishabh Saxena, Marvin Manz, Wenlan Liu, Jacob T. Blaskovits, Denis Andrienko, Jasper J. Michels, Paul W. M. Blom, Gert-Jan. A. H. Wetzelaer

**Affiliations:** Max Planck Institute for Polymer Research, Mainz, Germany

**Keywords:** Organic LEDs, Electronics, photonics and device physics

## Abstract

Blue organic light-emitting diodes based on thermally activated delayed fluorescence suffer from low stability and broad emission. Hyperfluorescence—in which the excited state created on the thermally activated delayed fluorescence emitter is transferred to a fluorescent terminal emitter with a narrow emission spectrum—is promising towards improving colour purity and stability. However, direct charge trapping on the smaller-gap terminal emitter may lead to direct emissive losses, inhibited charge transport and charge imbalance. Here we demonstrate single-layer pure-blue hyperfluorescent organic light-emitting diodes that are not compromised by charge trapping on the terminal emitter. We reveal that the energetic disorder of the thermally activated delayed fluorescence sensitizer allows for the presence of a terminal emitter with a smaller energy gap, without affecting charge transport. Consequently, the stability benefits of single-layer organic light-emitting diodes can be combined with trap-free hyperfluorescence, resulting in pure-blue emission, a simple device structure, high quantum and power efficiencies, and state-of-the-art operational stability.

## Abstract (web summary)

Single-layer organic light-emitting diodes based on thermally activated delayed fluorescence are demonstrated, exhibiting pure-blue emission, high quantum and power efficiencies, and operational stability.

## Main

Even though organic light-emitting diodes (OLEDs) are an established technology used in high-end displays, blue OLEDs are still not up to par with their green and red counterparts. Blue fluorescent emitters as used in displays exhibit decent operational stability, but their efficiency is limited by the fact that only singlet excitons are emissive. Blue phosphorescent emitters are more efficient as a result of triplet harvesting, but the operational stability of blue phosphorescent OLEDs remains low^1,2^. During the past decade, thermally activated delayed fluorescence (TADF) has emerged as a promising alternative to phosphorescence for harvesting triplet excitons^3^. However, as is the case for phosphorescent OLEDs, the operational stability of blue-emitting TADF devices is still relatively poor^4^. Moreover, TADF emitters usually exhibit a broad emission spectrum, compromising the colour purity of the OLED^5–7^. A strategy to overcome this issue is to add a low concentration of a terminal fluorescent emitter with a narrow spectrum, which is excited by energy transfer from the TADF emitter. This concept is usually referred to as hyperfluorescence^8^. However, the addition of a fluorescent emitter also involves potential energy loss mechanisms. In recent research, most of the attention has been focused on preventing Dexter energy transfer of triplet excitons from the TADF sensitizer to the triplet state of the fluorescent emitter, which is non-emissive^9–12^. Strategies to overcome this issue are the use of a low concentration of a fluorescent emitter (typically in the 1%–2% range) to diminish the possibility of short-range Dexter transfer, and the use of TADF emitters with a high reverse intersystem crossing rate to reduce the triplet-exciton population^10^.

Another less well investigated, but potentially more severe, loss mechanism is direct charge trapping on the terminal fluorescent emitter. As the fluorescent emitter is required to have a smaller energy gap than the TADF sensitizer, to allow energy transfer, it seems inevitable that the fluorescent emitter must be a trap for either electrons or holes, or both. Charge trapping can lower the current through organic semiconductors by several orders of magnitude, even when the concentration of trap states is as low as 0.1% (ref. ^13^). This implies that even a low amount of a smaller-gap fluorescent emitter can drastically inhibit charge transport^14,15^. Apart from reduced transport, the exclusive trapping of either electrons or holes can also induce charge imbalance in an OLED^16^. In addition, charge trapping on a fluorescent emitter can result in trap-assisted recombination, which results in 75% of the formed excitons, being in the triplet state, decaying non-radiatively^17^. Trap-assisted recombination dominates over bimolecular recombination even for a small relative trap concentration of 0.01%–0.1% (ref. ^18^). In hyperfluorescent OLEDs, the doping concentration is typically in the 1% range, which corresponds to a trap concentration of approximately 10^25^ m^−3^. Such a high trap concentration would greatly overwhelm the free charge carrier concentration of 10^22^–10^23^ m^−3^ in an OLED, implying that all charges would be effectively trapped.

Charge trapping as a loss process is usually not easily investigated, as common multilayer OLEDs comprise a multitude of charge transport layers and heterojunctions, which makes it difficult to isolate charge-trapping phenomena in the emissive layer^19–21^. Therefore, the effect of charge trapping in hyperfluorescent OLEDs is still poorly understood. Recently, we have demonstrated highly efficient and stable OLEDs based on TADF in a single-layer architecture, where the emissive layer is simply sandwiched between two ohmic contacts^22^. As a result, charge transport and recombination are completely controlled by a single layer, which is not only an advantage from a design and fabrication perspective but also allows for a more straightforward analysis of the device physics. Single-layer OLEDs have shown to be a promising alternative to conventional multilayer OLEDs, with similar efficiency, lower operating voltage and an inherently increased lifetime due to a broadened recombination zone. A major open question is if these advantages of single-layer OLEDs can also be transferred to hyperfluorescent OLEDs with a narrow emission spectrum, given the fundamental complications arising from charge trapping on the additional smaller-gap terminal emitter, compounded by the fact that charge transport is completely controlled by the emissive layer.

Here we demonstrate highly efficient and stable pure-blue single-layer OLEDs, based on hyperfluorescence. By virtue of the single-layer architecture, we are able to reveal that the addition of a terminal blue emitter does not result in additional charge trapping, despite its smaller energy gap than the TADF sensitizer. This paradoxical result is explained by considering the energetic disorder of the transport states, which effectively lowers the trap depth and, in this case, eliminates trapping completely. As a result, the terminal emitter does not affect the device performance of single-layer OLEDs other than converting the broad sky-blue emission spectrum of the TADF emitter into a narrow pure-blue emission peak. The simple single-layer OLED shows a high external quantum efficiency (EQE) of 25%, a high power efficiency (PE) at high brightness and an operational stability rivalling the state of the art for hyperfluorescent blue OLEDs.

**Fig. 1:**
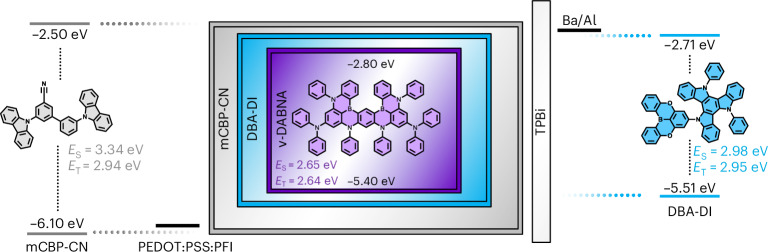
Device layout of a pure-blue hyperfluorescent single-layer OLED. The emissive layer comprising evaporated DBA-DI, mCBP-CN and 2% ν-DABNA is sandwiched between a PEDOT:PSS:PFI bottom anode and a barium/aluminium top cathode. To facilitate electron injection, a thin TPBi (3 nm) interlayer between the emissive layer and top contact is inserted, which does not exhibit any charge- or exciton-blocking functionality. The effective Fermi levels of the electrodes and the energy levels of DBA-DI, mCBP-CN and ν-DABNA are indicated^23,24,26^. In addition, the energy of the excited singlet (*E*_S_) and triplet (*E*_T_) states are indicated.

As the first step, we have selected 5-(5,9-dioxa-13*b*-boranaphtho[3,2,1-*de*]anthracen-7-yl)-10,15-diphenyl-10,15-dihydro-5*H*-diindolo[3,2-*a*:3′,2′-*c*]carbazole (DBA-DI) as the sky-blue TADF emitter, which has shown promising lifetimes in multilayer OLEDs due to its high reverse intersystem crossing rate, good electrochemical stability and high bond dissociation energy (BDE)^23^. Paired with the stable 3,3′-di(carbazol-9-yl)-5-cyano-1,1′-biphenyl (mCBP-CN) host, high EQEs have been achieved, although the electroluminescence (EL) spectrum is still relatively broad, as is common for TADF emitters^24^. The energy levels for this system is displayed in Fig. 1.

**Fig. 2:**
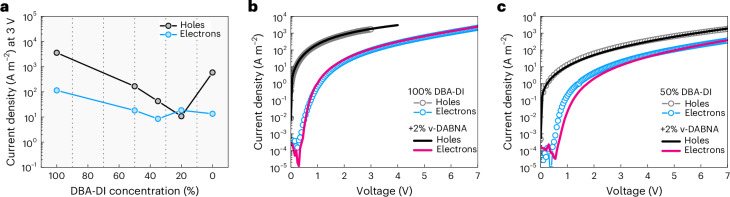
Charge transport in the emissive layer. **a**, Electron and hole current densities at a voltage of 3 V as a function of DBA-DI concentration in an mCBP-CN host, with film thicknesses in the 70–80-nm range. In the neat DBA-DI film, the transport is highly hole dominated. With a decreasing concentration of DBA-DI in mCBP-CN, charge transport becomes more balanced, with an optimum in the 20%–50% range. **b**, Electron and hole current densities versus voltage for pure DBA-DI (symbols) and for DBA-DI doped with 2% ν-DABNA (lines). **c**, Electron and hole current densities versus voltage for DBA-DI:mCBP-CN (1:1) (symbols) and for the host–guest system doped with 2% ν-DABNA (lines). Source data

To assess the viability of the mCBP-CN:DBA-DI host–guest system for utilization in a single-layer OLED, we first investigate the charge transport properties. As shown in Fig. 2a, the transport in pure DBA-DI is hole dominated, which is common for organic semiconductors with these energy levels^25^. As expected from the ionization energy offset, doping DBA-DI in an mCBP-CN matrix results in a continuous decrease in the hole transport with decreasing DBA-DI content, demonstrating that hole transport is dominated by guest–guest transport via DBA-DI. The electron transport, on the other hand, is less affected by a decreased DBA-DI concentration, implying that mCBP-CN assists in electron transport. Overall, charge transport becomes more balanced when doping DBA-DI in mCBP-CN. An optimum in terms of charge balance is observed for a DBA-DI concentration in the range of 20%–50%.

As DBA-DI still has a relatively broad emission spectrum (typical for most TADF emitters), we utilize an additional blue emitter with a narrow spectrum, which is excited via DBA-DI by energy transfer. For this purpose, we have selected the multiresonance TADF emitter ν-DABNA, which is a common terminal emitter in hyperfluorescent devices^26–28^. To allow energy transfer, the terminal emitter should have a smaller energy gap than the TADF sensitizer (in this case, DBA-DI). Energy transfer is confirmed by photoluminescence (PL) in the doped film (Supplementary Fig. 1), confirming the smaller energy gap of ν-DABNA compared with DBA-DI. However, a fundamental problem to be expected in this design is that the injected charges will preferably populate the smaller-gap emitter via charge trapping, leading to higher operating voltages, charge imbalance and direct trap-assisted recombination. Previous research has shown that charge trapping is very effective even in the 0.1% concentration range^13^, which is lower than the typical concentrations of terminal emitters used in hyperfluorescent OLEDs. For example, Supplementary Fig. 2 shows that the addition of only 1% ν-DABNA lowers the hole current through a 4,4′-bis(*N*-carbazolyl)-1,1′-biphenyl matrix by three orders of magnitude due to strong charge trapping.

An important question, therefore, is whether the addition of ν-DABNA would lead to additional charge trapping in the DBA-DI:mCBP-CN system and if so, render the fabrication of efficient single-layer hyperfluorescent OLEDs impossible. As shown in Fig. 2b,c, surprisingly, the addition of 2% ν-DABNA has a negligible impact on the measured electron and hole currents, showing that there is no charge trapping on ν-DABNA, despite its smaller energy gap. To resolve this paradoxical result, we have to consider the energetic disorder of the transport states. From temperature-dependent charge transport measurements (Supplementary Fig. 4), we infer a width *σ* of the density-of-states (DOS) distribution of 0.14 eV for electrons and 0.12 eV for holes, by fitting the electron and hole currents with drift-diffusion simulations. These values are obtained within the framework of the extended Gaussian disorder model, in which the temperature activation of mobility is controlled by the width of the energetic distribution of transport states^29^.

**Fig. 3:**
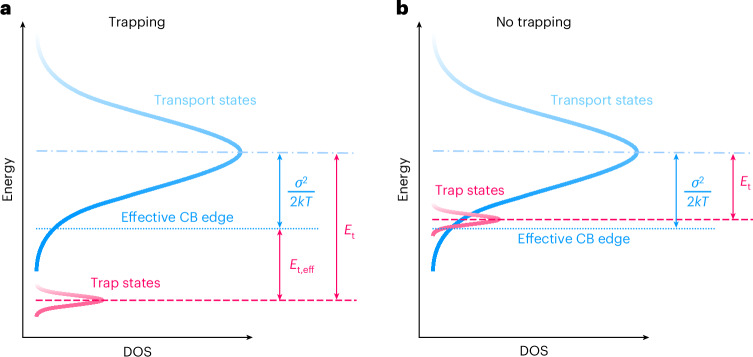
Schematic of the effect of energetic disorder on the trap depth. The Gaussian distribution of transport states (for electrons) is depicted in blue, with the effective conduction band (CB) edge (dotted line) at an energy *σ*^2^/2*kT* below the centre (dash–dotted line). **a**, Trap states (magenta) are located at an energy *E*_t_ below the mean of the distribution of transport states, corresponding to an effective trap depth *E*_t,eff_ with respect to the CB edge. **b**, For *E*_t_ ≤ *σ*^2^/2*kT*_,_ charges will not be trapped, since *E*_t,eff_ ≤ 0 in this case. The density of trap states is exaggerated for display purposes.

The broadened distribution of transport states allows for the presence of an emitter with a smaller energy gap, without affecting the charge transport (Fig. 3). To understand to what extent energetic disorder can prevent charge trapping on a terminal emitter, it is useful to consider the effective conduction band or valence band edge energy for the case of a Gaussian-distributed DOS. In this case, the effective trap depth *E*_t,eff_ is lowered by *σ*^2^/2*kT* compared with the absolute trap depth *E*_t_, which is the energy of the trap with respect to the centre of the Gaussian DOS distribution (Fig. 3a)^30^. In case of sufficiently shallow traps, that is, *E*_t_ ≤ *σ*^2^/2*kT*, the effective trap depth is smaller than zero, implying that it is no longer energetically favourable and, hence, does not trap charges (Fig. 3b). For DBA-DI, *σ*^2^/2*kT* equals 0.28 eV for holes and 0.38 eV for electrons. The energy levels of ν-DABNA fall well within these margins, and thus, the effective trap depth equals zero. As a result, for hyperfluorescent OLEDs, there is a considerable margin—here up to more than 0.6 eV—for the presence of a smaller-gap emitter, without affecting the charge transport.

**Fig. 4:**
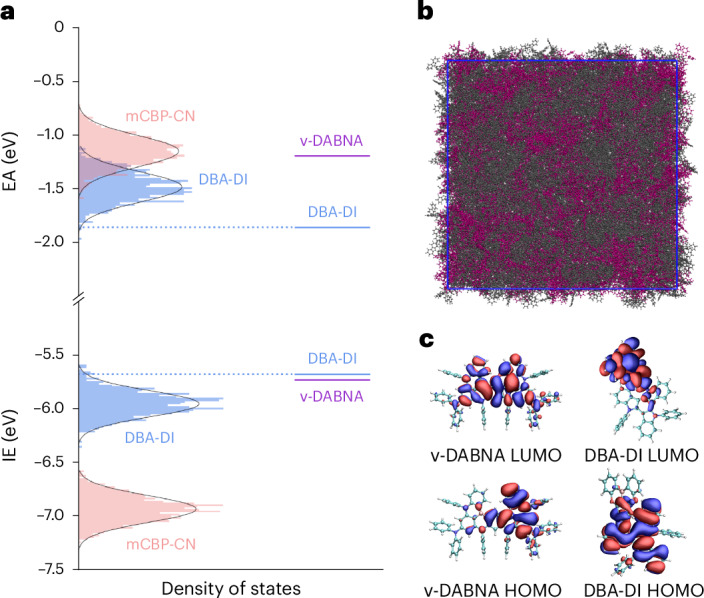
Simulated DOS distributions of the emissive layer. **a**, Solid-state distributions of DOS for the DBA-DI:mCBP-CN simulated morphology are represented as histograms, with a Gaussian fit displayed by the black solid lines. The solid blue lines represent the onset of DBA-DI IE and EA distributions, which are offset from the mean of the distribution by *σ*^2^/2*kT*. The IE and EA values of ν-DABNA in an implicit solvent are indicated by the solid violet lines. **b**, Simulation snapshot of the mCBP-CN:DBA-DI system, where the mCBP-CN host is shown in grey and the TADF emitter DBA-DI is shown in magenta. **c**, Omega-tuned frontier orbitals of the TADF emitters DBA-DI and ν-DABNA. HOMO, highest occupied molecular orbital; LUMO, lowest unoccupied molecular orbital. Source data

To evaluate this energetic picture in more detail, we ran multiscale simulations of the DBA-DI:mCBP-CN mixture (Fig. 4). The DOS values of mCBP-CN and DBA-DI in a mixed host–guest system, with a 40% weight fraction of DBA-DI, were computed using VOTCA (Supplementary Information)^31^. From these simulations, the energy distributions of ionization energy (IE) and electron affinity (EA) of the host and guest reveal that hole transport is expected to be exclusively carried by DBA-DI, whereas the close vicinity between the energetic position of the EA distributions reveal that electron transport may be partially assisted by mCBP-CN. These observations are in line with the experimental concentration-dependent charge transport characteristics (Fig. 2a). Furthermore, the simulated width of the DBA-DI DOS is 0.14 eV for electrons and 0.12 eV for holes, in close agreement with the experimental results. Importantly, the energy levels calculated for ν-DABNA in a dielectric medium fall within the distribution of DBA-DI transport states, rationalizing the absence of charge trapping on this emitter. Since organic semiconductors used in OLEDs commonly exhibit energetic disorder, this result is of general relevance for hyperfluorescent OLEDs.

**Fig. 5:**
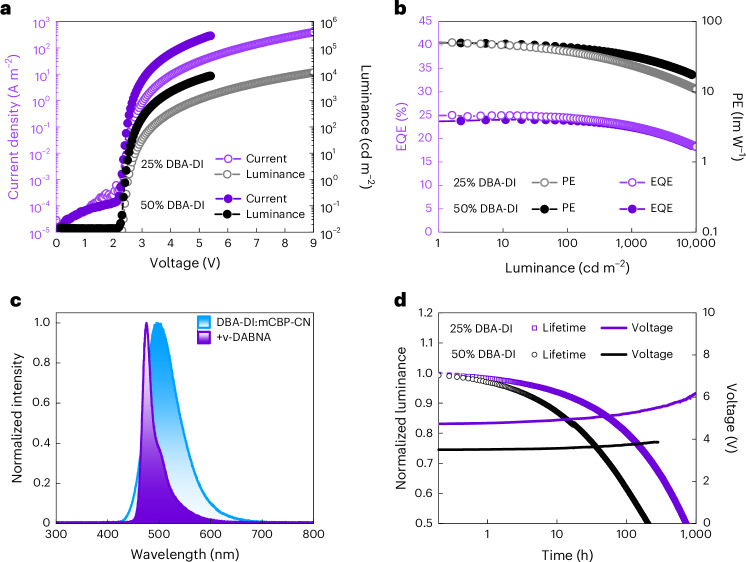
Device performance of single-layer pure-blue hyperfluorescent OLEDs. **a**, Current density–voltage and luminance–voltage characteristics for ν-DABNA:DBA-DI:mCBP-CN OLEDs, either with 25% DBA-DI or 50% DBA-DI, with film thicknesses of 81 nm and 82 nm, respectively. **b**, Corresponding EQE and PE versus luminance. **c**, Electroluminescence spectra of the hyperfluorescent OLEDs with a maximum at 475 nm and FWHM of 22 nm compared with a DBA-DI:mCBP-CN OLED without ν-DABNA. **d**, Operational lifetimes of pure-blue hyperfluorescent OLEDs at an initial luminance of 1,000 cd m^−2^, with the normalized luminance (symbols) and driving voltage (lines) as a function of operation time at constant current. Source data

The fact that charge transport is unaffected by doping the DBA-DI:mCBP-CN matrix with ν-DABNA shows promise for hyperfluorescent single-layer OLEDs, in which charge transport is entirely controlled by the emissive layer. For OLEDs, we use poly(3,4-ethylenedioxythiophene) polystyrene sulfonate (PEDOT:PSS):perfluorinated ionomer (PFI) as an ohmic high-work-function hole contact (Supplementary Fig. 5), and TPBi (3 nm)/Ba/Al as an electron contact^32–34^, giving the device structure shown in Fig. 1. The 3-nm TPBi layer solely functions as a tunnelling interlayer for charge injection (Supplementary Fig. 6), as it is too thin to contribute to the electron transport or have any hole- or exciton-blocking functionality^35,36^. As a result, the OLED has a single-layer architecture from an operational perspective, without any charge confinement by blocking layers, with the recombination zone well separated from the electrode interfaces due to balanced charge transport. In Fig. 5a,b, the current density–voltage and luminance–voltage characteristics are shown, respectively, for single-layer hyperfluorescent OLEDs, for DBA-DI concentrations of 25% and 50%.

The electroluminescence spectra (Fig. 5c) show near-complete energy transfer from DBA-DI to ν-DABNA, as evidenced by the narrowband blue emission peak at 475 nm with a full-width at half-maximum (FWHM) of 22 nm. The single-layer OLEDs exhibit high maximum EQEs of 24%–25% with very limited roll-off until 1,000 cd m^−2^, where the EQE remains at 22%–23%. Both OLEDs demonstrate remarkably low turn-on voltages of 2.5 V (25% DBA-DI) and 2.4 V (50% DBA-DI), determined at 1 cd m^−2^. These values are exceptionally low for pure-blue light emission and are even lower than the optical bandgap of the emissive molecule ν-DABNA (2.63 eV), which is attributed to diffused charge carriers below the built-in voltage^37^. In particular, a luminance of 100 cd m^−2^ for the device with 50% DBA-DI is already reached at 2.84 V (25% DBA-DI: 3.35 V) and 1,000 cd m^−2^ at 3.55 V (25% DBA-DI: 4.90 V). The reason for the lower operating voltage of the OLED with a higher DBA-DI loading is the better overall charge transport, which mainly takes place via DBA-DI.

The maximum PE values range between 50 lm W^−1^ and 51 lm W^−1^, which are among the highest reported values for a blue hyperfluorescent OLED incorporating a terminal emitter with a small FWHM^8,28,38–41^.

The device with 50% DBA-DI maintains impressive PE values of 43 lm W^−1^ at 100 cd m^−2^ and 32 lm W^−1^ at 1,000 cd m^−2^, being twice as high as reference devices incorporating ν-DABNA (Table 1)^8,26^. These high PE values are the result of the low operating voltages due to the absence of heterojunctions in the single-layer architecture. Considering these results, efficient pure-blue hyperfluorescent single-layer OLEDs are feasible, with efficiencies that rival more complex multilayer devices.

**Table 1:** Summary of device characteristics of the hyperfluorescent OLEDs and comparison to reference devices

	Voltage (V)^a^	EQE (%)^b^	PE (lm W^−1^)^c^	CE (cd A^−1^)^d^	CIE(*x*, *y*)	LT_80_/LT_50_ (h)^e^
25% DBA-DI	2.5/3.4/4.9	25/24/23	51/37/24	41/40/37	(0.13, 0.27)	110/716
50% DBA-DI	2.4/2.9/3.6	24/24/22	50/43/32	40/39/36	(0.12, 0.27)	25/205
Ref. 1 (ref. ^8^)	3.0/4.2/5.6	27/24/20	41/26/16	39/36/31	(0.15, 0.20)	~90/–
Ref. 2 (ref. ^26^)	3.4/−/−	34/33/26	26/21/12	31/30/23	(0.12, 0.11)	<3/31^f^
Ref. 3^g^	7.4/11.5/14.7	9/9/8	6/4/3	15/14/13	(0.15, 0.24)	–

^a^Voltage value at 1/100/1,000 cd m^−2^

^b^EQE at maximum/100/1,000 cd m^−2^

^c^PE at maximum/100/1,000 cd m^−2^

^d^Current efficiency (CE) at maximum/100/1,000 cd m^−2^

^e^Determined at an initial luminance of 1,000 cd m^−2^

^f^Determined at an initial luminance of 100 cd m^−2^

^g^Reference device without DBA-DI as the sensitizer (Supplementary Fig. 8).

When comparing the OLED characteristics to the control devices without ν-DABNA (Fig. 5c and Supplementary Fig. 7), it is evident that the addition of ν-DABNA only affects the electroluminescence spectrum, which shifts from a broader sky-blue spectrum to a narrowband blue emission peak, with similar current density–voltage and luminance–voltage characteristics. This again confirms that ν-DABNA is excited by energy transfer from DBA-DI, without causing charge trapping and, therefore, changing the electrical characteristics. The EL spectrum is also identical to the PL spectrum (Supplementary Fig. 1), in accordance with energy transfer being the sole excitation mechanism. By contrast, for the ν-DABNA control device without DBA-DI (Supplementary Fig. 8), the current density is severely suppressed, which is caused by hole trapping on ν-DABNA, having a much lower IE than mCBP-CN. Direct trapping and recombination on ν-DABNA leads to a reduced EQE, whereas the compromised charge transport due to trapping results in substantially higher operating voltages and lower PE values (Table 1). This exemplifies the problem of charge trapping when using a small concentration of a smaller-gap emitter, which can be successfully mitigated when utilizing the energetic disorder of the TADF sensitizer.

Apart from the broad electroluminescence spectra commonly observed for TADF emitters, another main issue for TADF OLEDs is limited stability, especially for blue TADF emitters. A single-layer OLED structure fundamentally reduces interactions between excitons and polarons, due to the broadened recombination zone and the absence of blocking layers^42^, where the latter can lead to high charge and exciton concentrations near a blocking interface. Therefore, the single-layer architecture offers a promising route to more stable OLEDs. Indeed, as shown in Supplementary Fig. 9, the non-hyperfluorescent single-layer DBA-DI:mCBP-CN OLED exhibits a lifetime longer than a reported multilayer OLED based on the same host–guest system. Our single-layer hyperfluorescent devices with ν-DABNA exhibit an impressive LT_50_ of 716 h (25% DBA-DI) at an initial luminance of 1,000 cd m^−2^ (Fig. 5d), comparable with state-of-the-art blue hyperfluorescent OLEDs.

The lifetimes of hyperfluorescent OLEDs are very similar compared with their non-hyperfluorescent, DBA-DI:mCBP-CN-based counterparts for different DBA-DI concentrations (Supplementary Fig. 9). This is expected considering that OLED degradation is believed to be mainly driven by the interactions between triplet excitons and polarons. As the terminal emitter ν-DABNA is excited by the energy transfer of singlet excitons from DBA-DI, without participating in charge transport, the triplet and polaron concentrations in the DBA-DI:mCBP-CN matrix remain practically unaffected, which gives rise to a similar degradation behaviour of the TADF and hyperfluorescent OLEDs. This is also in line with the unaffected charge transport (Fig. 2b,c) and EQE characteristics by the inclusion of ν-DABNA, which all underline that the sole effect of ν-DABNA is the spectral conversion via singlet energy transfer, confirming trap-free hyperfluorescence.

To gain more insights in the origin of the high stability of this system, we considered several variations in terms of materials and device composition. First, it is evident that the lifetime is strongly dependent on the DBA-DI concentration (Supplementary Fig. 9), with pure DBA-DI OLEDs having the shortest lifetime, indicating that the stability of DBA-DI plays a crucial role. It is experimentally shown that DBA-DI is more stable towards holes than towards electrons (Supplementary Fig. 10). Correspondingly, the calculated BDE values for DBA-DI are the lowest in the anionic state (Supplementary Fig. 15). By using mCBP-CN as an electron-transporting host, electrons are partly shared by the host, leading to higher operational stability with increasing host content (Supplementary Fig. 9). Conversely, when using mCBP as a host, which does not participate in electron transport due to its low EA, the lifetime is drastically reduced to only 9 h (Supplementary Fig. 13). A similar lifetime is observed for DBA-DI in an mCPCN host, which also shows hole-dominated transport (Supplementary Fig. 11). By contrast, with the electron-transporting host SiTrzCz2, the electron–hole balance increases (Supplementary Fig. 12), accompanied by enhanced operational stability. The stability of the terminal emitter ν-DABNA does not seem to limit the lifetime in our devices, even though the BDE of ν-DABNA in the anionic state is slightly lower than DBA-DI. This would be in line with ν-DABNA being excited purely via energy transfer, rather than via electron trapping.

Although it is clear that the choice of host material manipulates the lifetime by means of charge distribution on the sensitizer, it is not completely clear how the stability of the host itself affects the lifetime. For example, bond dissociation in the host may not directly affect radiative recombinations on the emitter. When moving to different sensitizers, one has to additionally consider the excited-state dynamics. As an example, two recently reported ν-DABNA-based hyperfluorescent OLEDs with an mCBP-CN host exhibited vastly different lifetimes based on the used sensitizer^43^. Although this difference is in part reflected in the BDE (Supplementary Fig. 15), the operational stability for both these sensitizers is clearly lower than DBA-DI as the sensitizer, which has short excited-state lifetimes. Likewise, our previously reported blue TADF single-layer OLEDs exhibit short operational lifetimes (Supplementary Fig. 14), although the anion BDE of the emitter is higher than DBA-DI, indicating that the excited-state dynamics are an important factor in the operational stability^32^. Overall, it can be concluded that the stabilities of both sensitizer and host, as well as their respective energy alignment, are key requirements for achieving long-lifetime TADF OLEDs. Additional avoidance of charge trapping on the terminal emitter, as demonstrated here, ensures the conservation of operational stability, efficiency and charge balance.

In conclusion, we have demonstrated trap-free hyperfluorescence, to yield highly efficient and stable pure-blue single-layer OLEDs, operating at low voltages. By means of charge transport measurements, it is shown that the terminal emitter does not induce charge trapping, despite its smaller energy gap. This paradoxical result is rationalized by considering the energetic disorder of the DOS of the TADF sensitizer and host, which effectively disables charge trapping on the terminal emitter. Therefore, energetic disorder plays an important role in hyperfluorescent OLEDs, and should be taken into account in their design. The absence of charge trapping enables combining hyperfluorescence with single-layer OLEDs, exploiting their inherent stability advantages. In these highly simplified OLEDs, the broad sky-blue emission spectrum of the TADF sensitizer is converted to a narrow pure-blue emission spectrum via singlet energy transfer, without comprising EQE or lifetime. The limiting factor in the lifetime is shown to be the stability of the sensitizer in the anionic state, which can be improved by utilizing an electron-transporting host to distribute the electrons. As a result of trap-free hyperfluorescence, simple single-layer OLEDs with pure-blue emission, high efficiency and long lifetime are demonstrated.

## Methods

### Materials

Nafion™ (PFI) was purchased from Sigma-Aldrich as a 5 wt% solution in a mixture of lower aliphatic alcohols and water, containing 45% water. DBA-DI and ν-DABNA were purchased from Luminescence Technology in the sublimed grade. mCBP-CN, TPBi, C_60_, CBP, mCBP, mCPCN and SiTrzCz2 were purchased from Ossila BV in sublimed grade.

### Device fabrication

Substrates prepatterned with indium tin oxide were cleaned by washing with a detergent solution and ultrasonication in acetone (5 min) and subsequently in isopropyl alcohol (5 min), followed by ultraviolet–ozone treatment (50 min). Dispersions for the hole injection layer were prepared 24 h before device fabrication by mixing PEDOT:PSS (CLEVIOS™ P VP AI 4083) with Nafion™ in a 1:6:14 ratio (PEDOT:PSS:PFI) and diluted in deionized water (1:1). PEDOT:PSS:PFI was applied by spin coating, resulting in films of 20-nm thickness, which were subsequently annealed at 130 °C for 12 min. The substrates were then transferred to a nitrogen-filled glovebox. Thermal evaporation of the emissive layer was performed at a base pressure of 2–3 × 10^−6 ^mbar. TPBi (3 nm), barium (2.5 nm) and aluminium (100 nm) were evaporated to finalize the top contact.

For hole-only devices, a top contact consisting of C_60_ (4 nm), MoO_3_ (10 nm) and aluminium (100 nm) was evaporated.

For electron-only devices, aluminium (30 nm) was thermally evaporated on cleaned glass substrates, followed by the thermal evaporation of a layer of organic semiconductor. TPBi (3 nm), barium (2.5 nm) and aluminium (100 nm) were evaporated to finalize the device.

### Measurements

Electrical characterization was carried out under a nitrogen atmosphere with a Keithley 2400 source meter and the light output was recorded using a Si photodiode with NIST-traceable calibration. The photodiode was placed close to (but not in contact with) the OLED to capture all the photons emitted in the forward hemisphere. To avoid any light detection emitted from the substrate edges, the edges were masked by the sample holder, and the substrate size (3 × 3 cm^2^) was considerably larger than the photodetector area. The EQE, luminance and PE were calculated from the measured photocurrent, device current and electroluminescence spectrum. Electroluminescence spectra were obtained with a USB4000-UV-VIS-ES spectrometer.

### Computational simulations

The details and references for computational simulations (force-field parameterization, molecular dynamics simulations, DOS calculations, frontier orbital calculations and BDE computations) are given in the Supplementary Information.

## Online content

Any methods, additional references, Nature Portfolio reporting summaries, source data, extended data, supplementary information, acknowledgements, peer review information; details of author contributions and competing interests; and statements of data and code availability are available at 10.1038/s41563-025-02294-8.

## Supplementary information

Supplementary Information Supplementary Figs. 1–15, Table 1 and Details of computational simulations.

## Source data

Source Data Figs. 2, 4 and 5 Source data for Figs. 2, 4 and 5 that contain experimental or simulated data points.

## Data Availability

The data that support the plots within this paper are available via figshare at 10.6084/m9.figshare.29314397. Source data are provided with this paper.
