# Supplementary material for: Pure-blue single-layer organic light-emitting diodes based on trap-free hyperfluorescence
Source: Nat Mater. 2025 Jul 21;24(11):1742–8. doi: 10.1038/s41563-025-02294-8 (PMC12575368; doi:10.1038/s41563-025-02294-8)
Supplement: Supplementary file 1 — Supplementary Figs. 1–15, Table 1 and Details of computational simulations. [file 41563_2025_2294_MOESM1_ESM.pdf]

nature materials

Supplementary information https://doi.org/10.1038/s41563-025-02294-8

Pure-blue single-layer organic light-emitting diodes based on trap-free hyperfluorescence

In the format provided by the authors and unedited

Table of contents

1. Supporting experimental results
2. Bond-dissociation energy calculations
3. Details of computational simulations
4. References

1. Supporting experimental results

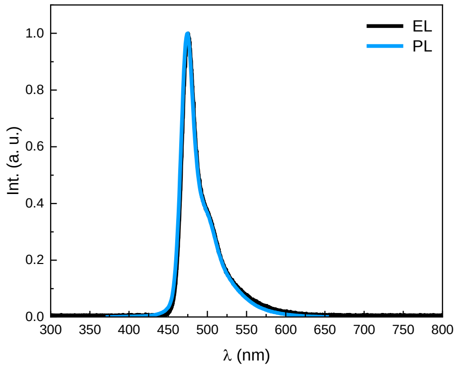

Figure S1. Comparison of steady state PL and EL of a DBA-DI:mCBP-CN (25:75) film with 2% v-DABNA doping demonstrating that v-DABNA is excited via energy transfer in electroluminescence.

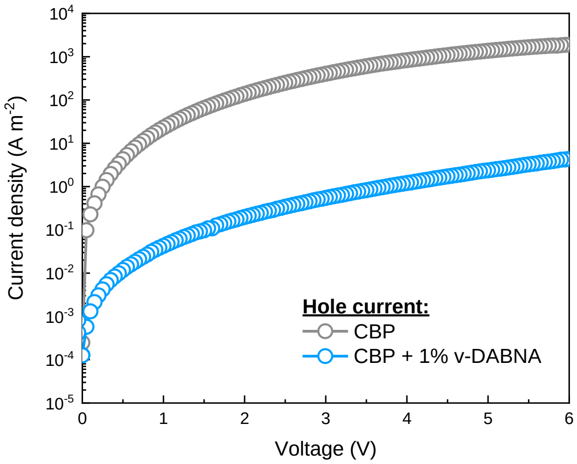

Figure S2. Hole current density vs voltage for a CBP hole-only device with and without 1% v-DABNA. The hole current is reduced by 3 orders of magnitude due to hole trapping on v-DABNA.

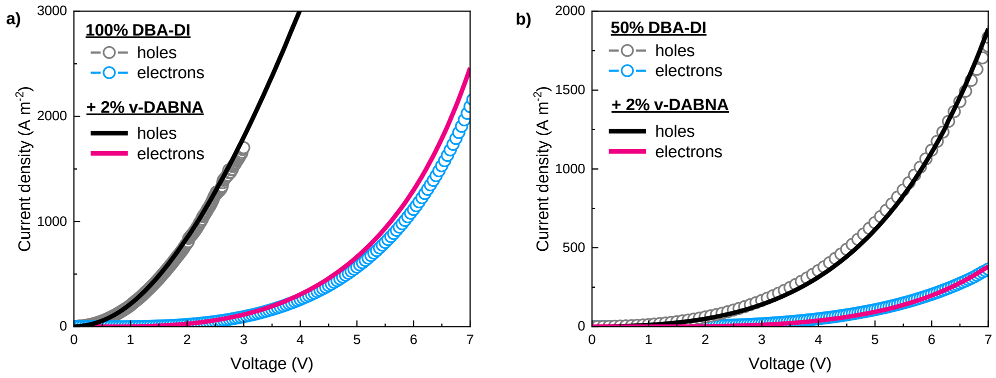

Figure S3. a) Linear-scale plots of the electron and hole current density vs voltage for pure DBA-DI (symbols) and for DBA-DI doped with 2% v-DABNA (lines). b) Electron and hole current density vs voltage for DBA-DI:mCBP-CN (1:1) (symbols) and for the host-guest system doped with 2% v-DABNA (lines).

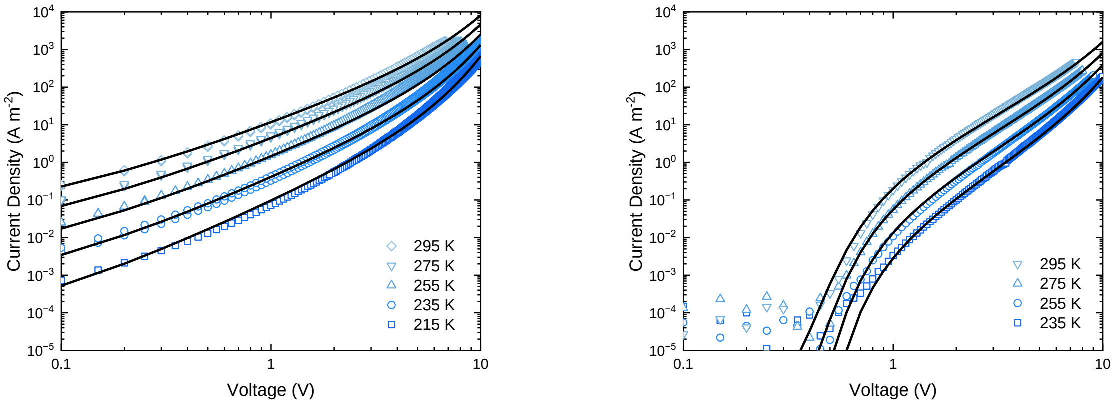

Figure S4. Temperature-dependent current density vs voltage characteristics of DBA-DI:mCBP-CN (1:1) hole-only (left) and electron-only devices (right). Symbols represent experimental data and solid lines are simulations with a numerical drift-diffusion solver, in which the temperature-, field-, and density-dependent mobility is described by the extended Gaussian disorder model, using a single set of parameters as given in Table S1. The hole-only device structure is ITO/PEDOT:PSS:PFI/DBA-DI:mCBP-CN(74 nm)/C_{60}(4 nm)/MoO_3(10 nm)/Al, the electron-only device structure is Al/DBA-DI:mCBP-CN(74 nm)/TPBi(3 nm)/Ba(2.5 nm)/Al.

Table S1. EGDM parameters as used in the drift-diffusion simulations of the current density vs voltage characteristics of the hole-only and electron-only devices. The simulated curves are provided in Fig. S2. The simulations were performed with an ohmic injecting contact. For the hole-only device the extracting contact is also ohmic (symmetric device), while for the electron contact a barrier of 0.8 eV is used at the extracting contact to account for the built-in voltage, which arises from the non-ohmic aluminum bottom contact in the electron-only device. A small number of traps is used in the simulations, which only has a minor effect on the low-voltage part of the *J-V* characteristics and are quickly filled by charge carriers.

	Holes	*Electrons*
Mobility prefactor μ_0	180 $m^2 V^{-1} s^{-1}$	350 $m^2 V^{-1} s^{-1}$
Energetic disorder σ	120 meV	140 meV
Lattice constant a	1.25 nm	1.2 nm
Mobility μ_0 (295 K)[a]	2.8×10^{-11} $m^2 V^{-1} s^{-1}$	1.8×10^{-12} $m^2 V^{-1} s^{-1}$
Trap density	1.7×10^{16} cm^{-3}	3×10^{16} cm^{-3}

[a] The mobility at 295 K is calculated from the mobility prefactor μ_0 and energetic disorder σ, as an example. The value represents the mobility at vanishing charge-carrier density and electric field.

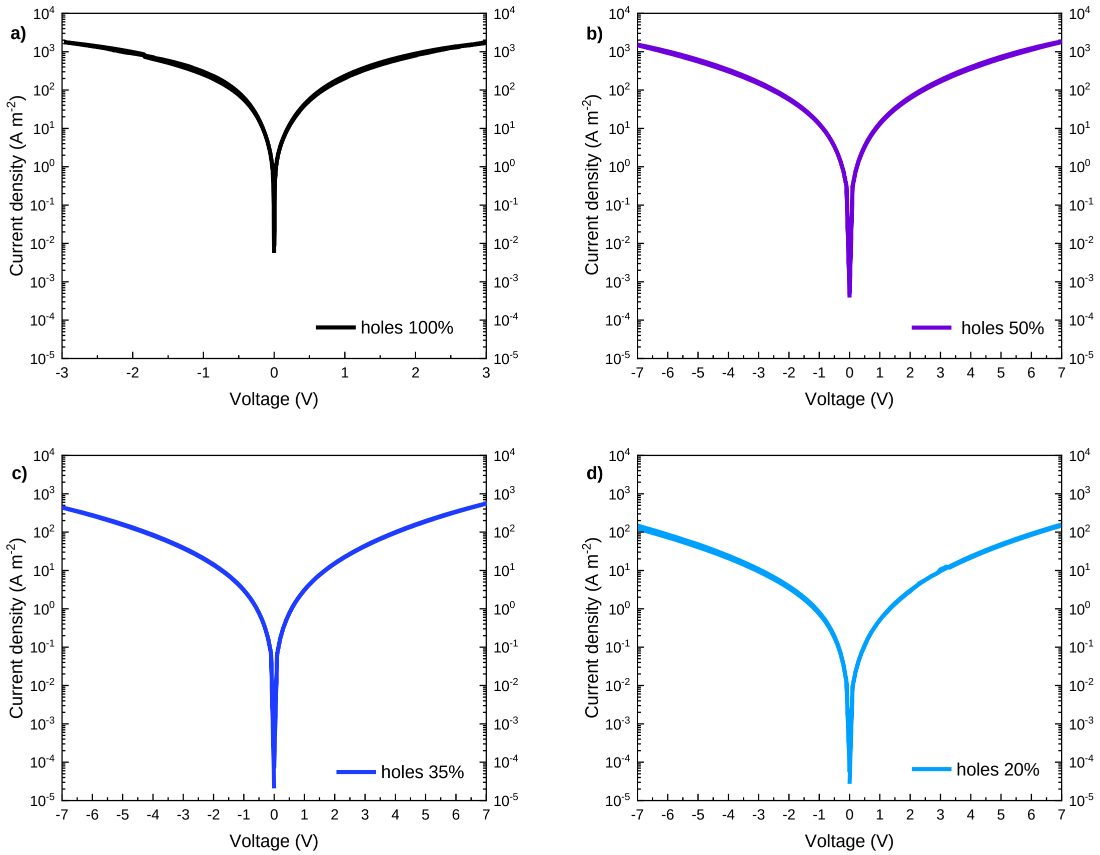

Figure S5. Current density-voltage characteristics of PEDOT:PSS:PFI/DBA-DI:mCBP-CN/C_{60}/MoO_3/Al hole only devices with different DBA-DI (100%, 50%, 35%, 20%) concentration. Negative bias corresponds to ohmic hole injection from the top contact comprising C_{60}/MoO_3 as the injection structure. Positive bias corresponds to hole injection from the bottom PEDOT:PSS:PFI layer. The perfectly symmetric characteristics demonstrate that PEDOT:PSS:PFI is an ohmic hole contact for all guest-host concentrations.

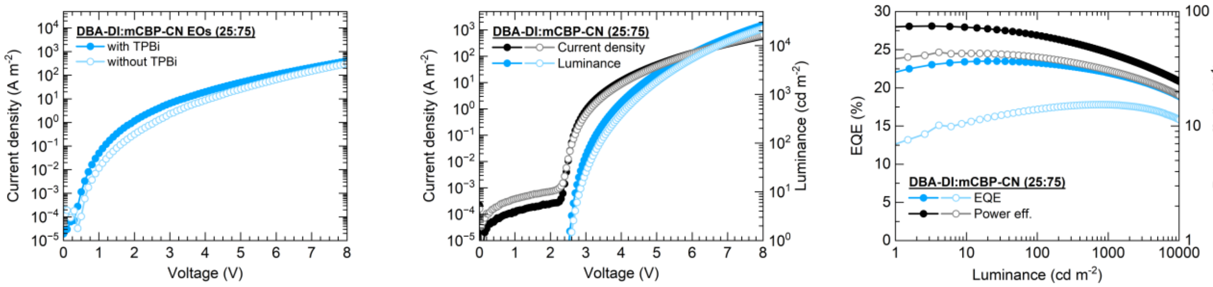

Figure S6. Comparison of DBA-DI:mCBP-CN (25:75, 78 nm) devices with (closed symbols) and without (open symbols) a 3 nm TPBi interlayer. a) electron-only device b) OLED JVL c)EQE and PE vs luminance. The electron current increases with the addition of the TPBi interlayer, showing that TPBi functions as an injection layer. Due to the non-ohmic contact without TPBi, the EQE shows a maximum of 18%, compared to 23.5% with TPBi interlayer.

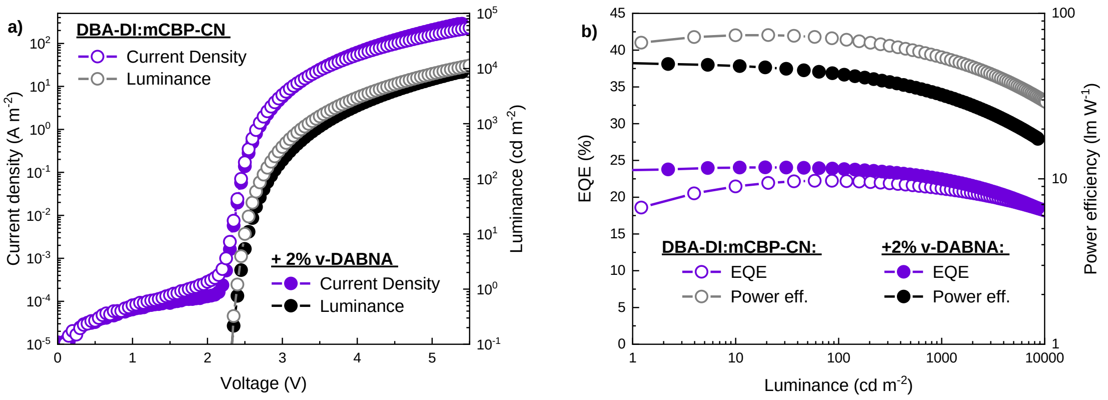

Figure S7. a) Current density-voltage-luminance characteristics of a DBA-DI:mCBP-CN (50:50) OLED with and without v-DABNA (2%) as a terminal emitter. The device structure is PEDOT:PSS:PFI (20 nm)/DBA-DI:mCBP-CN (74 nm)/TPBi (3 nm)/Ba (3 nm)/Al (100nm). b) corresponding EQE-L-PE plot. The current density is hardly affected by the addition of v-DABNA, confirming the absence of trapping.

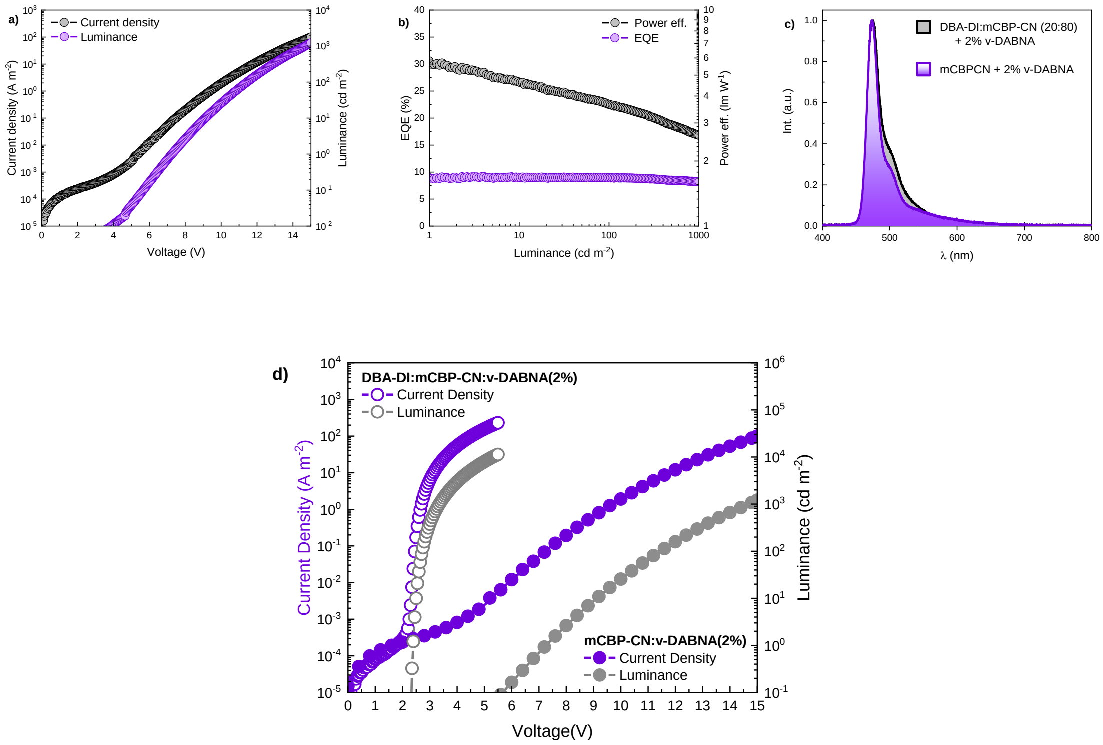

Figure S8. a) Current density-voltage-luminance characteristics of a v-DABNA:mCBP-CN OLED with 2% v-DABNA. The device structure was PEDOT:PSS:PFI (20 nm)/ v-DABNA:mCBP-CN (114 nm)/TPBi (3 nm)/Ba (3 nm)/Al (100nm). b) corresponding EQE-L-PE plot. c) electroluminescence spectra; peak: 474 nm, FWHM: 20 nm. Due to direct trapping and recombination, the spectrum is slightly narrowed compared to EL spectrum of the hyperfluorescent OLED with a DBA-DI sensitizer, which is excited via energy transfer, as confirmed by the PL spectrum in Fig. S1. d) Comparison between the *J-V-L* characteristics of (DBA-DI):mCBP-CN:v-DABNA(2%). Without DBA-DI, the current density is lowered by 5 orders of magnitude due to charge trapping on v-DABNA.

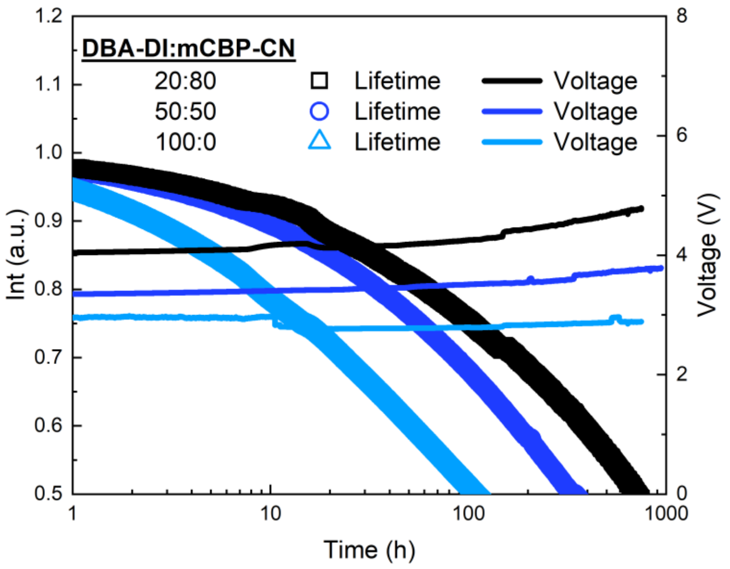

Figure S9. Operational lifetimes of non-hyperfluorescent blue OLEDs with varying DBA-DI (100%, 50%, and 20%) concentration. Normalized luminance (symbols) and driving voltage (lines) as a function of operation time determined at constant current in N_2 atmosphere with an initial luminance of 1000 cd m^{-2}.

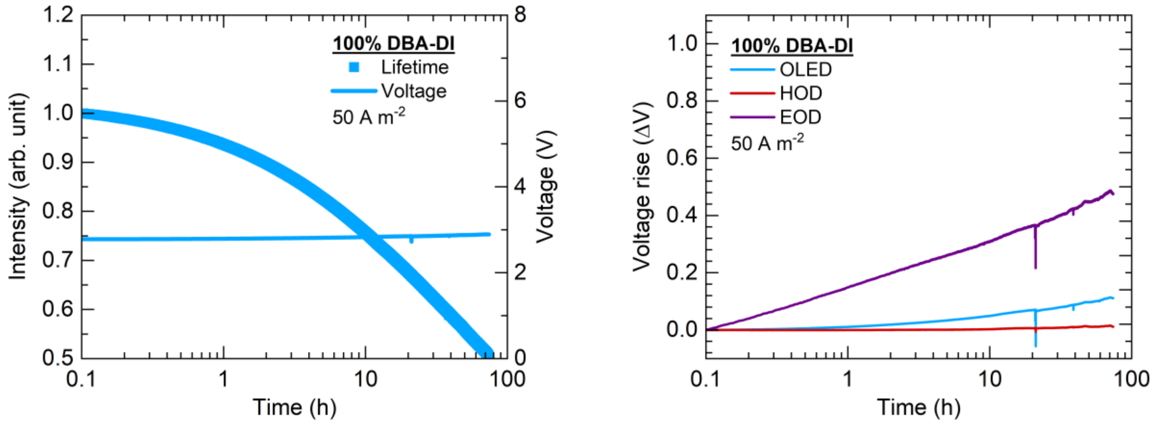

Figure S10. Degradation characteristics of pure DBA-DI devices (80 nm) stressed at a constant current density of 50 A m^{-2}. a) Normalized luminance (symbols) and driving voltage (line) of the OLED b) voltage rise of OLED, hole and electron-only devices. The voltage rise for the electron-only device is much stronger than the hole-only device, which indicates that the electron transport degrades faster. Since the OLED is hole-dominated for 100% DBA-DI, the OLED voltage rise is lower than for the electron-only device.

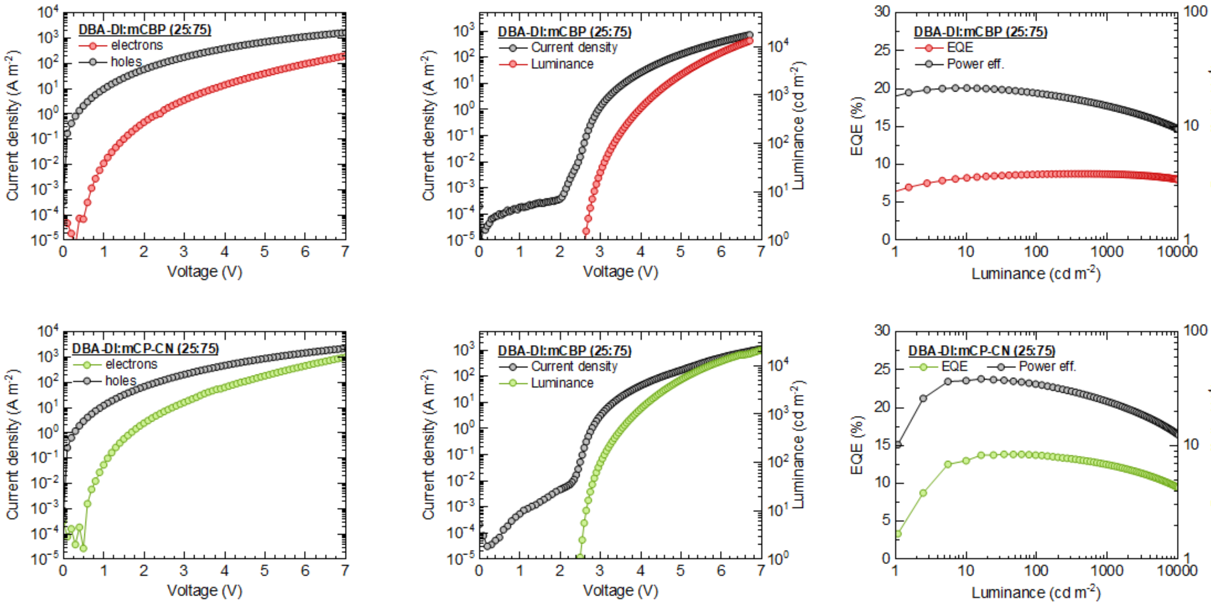

Figure S11. DBA-DI:mCBP (25:75) devices (top) and DBA-DI:mCPCN (25:75) devices (bottom). (left) hole and electron-only devices; (middle) OLED current density-luminance-voltage characteristics; (right) EQE and PE vs luminance. With both hosts, the charge transport remains hole dominated, even at relatively high host concentrations.

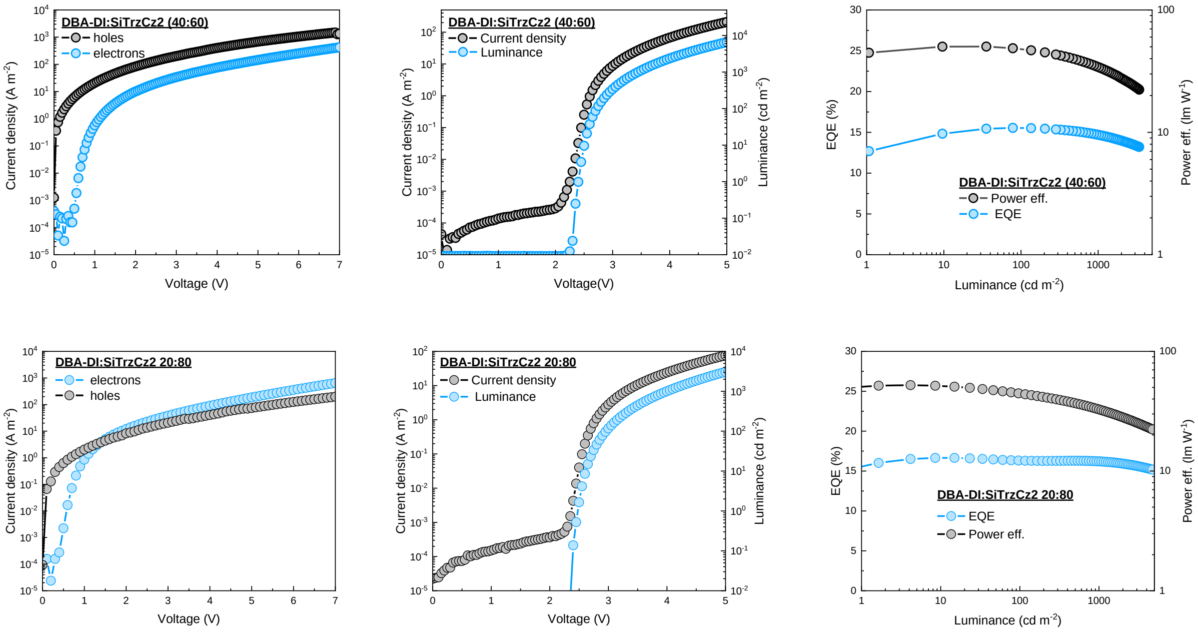

Figure S12. DBA-DI:SiTrzCz2 in 40:60 and 20:80 ratio. (left) hole and electron-only devices; (middle) OLED current density-luminance-voltage characteristics; (right) EQE and PE vs luminance. The n-type host SiTrzCz2 can shift the balance from hole-dominated to electron-dominated transport at increasing host concentration.

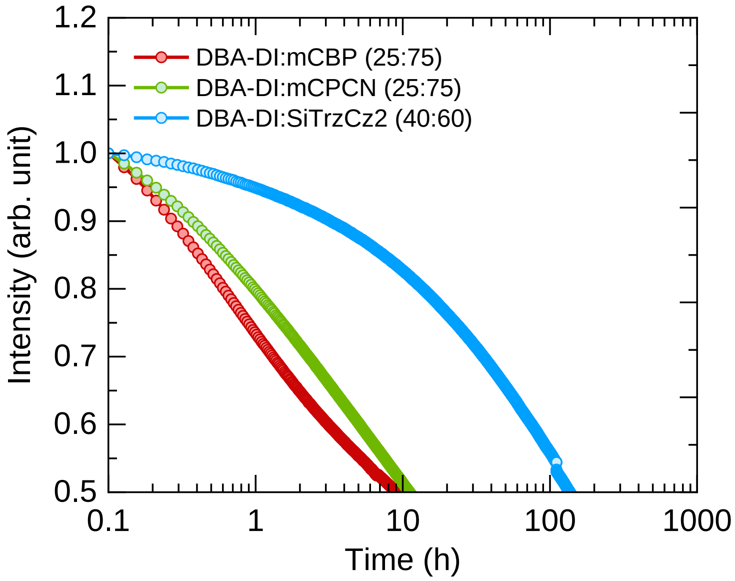

Figure S13. Operational lifetimes of DBA-DI TADF OLEDs with different hosts at an initial luminance of 1000 cd m^{-2}. The LT_{50} lifetimes are 9 hours for mCBP, 11 hours for mCPCN, and 137 hours for SiTrzCz2. Both mCBP and mCPCN have high LUMOs, electron transport takes place mostly on DBA-DI, resulting in short lifetimes. The SiTrzCz2 host assists in electron transport, giving rise to elongated operational lifetime, even at lower host ratio.

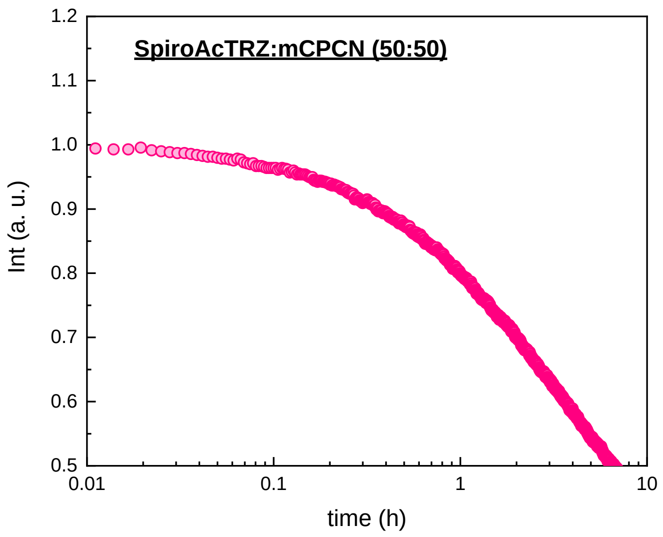

Figure S14. Lifetime of a SpiroAcTRZ:mCPCN OLED [PEDOT:PSS:PFI (20 nm)/SpiroAcTRZ:mCPCN (70 nm)/TPBI (4 nm) / Ba (3 nm)/ Al (100 nm)] stressed at 100 cd m^{-2} yielding an LT_{50} of 7 hours.

2. Bond-dissociation energy calculations

Bond dissociation energy (BDE) calculations: All DFT computations were performed with Gaussian 16 (Rev. B.01).[1] The geometries of all structures were optimized at the PBE0[2,3]–D3(BJ)[4–7]/def2-SVP[8] level. Frequency calculations confirmed that all stationary points were minima. Bond scans of the bonds in question for the C-N bonds of a reference system (DBA-DI) confirmed that the bond-breaking process did not involve a clear transition state. Therefore, the bond dissociation energy (BDE) was considered as the difference of free energy between the products of the dissociation and the initial relaxed structure. For the neutral state, the dissociation into two radical fragments (homolytic) and into both combinations of positively and negatively charged fragments were considered; in the charged states, both charged/radical fragment combinations were considered. For each state, the charge attribution leading to the lowest BDE is reported. In earlier work, it has been implicitly assumed that the electron added to the system in the anionic state remains on the 'acceptor' part of the donor-acceptor molecule (where the LUMO is located).[9] However, our results align with other studies[10] which show that a much lower BDE is obtained when the negative charge is assigned to the 'donor' part. In the case of DBA-DI, this corresponds to the negative charge being located on the core (rather than the multiresonant substituent), likely as a result of the higher electronegativity of the nitrogen atom on the broken bond.

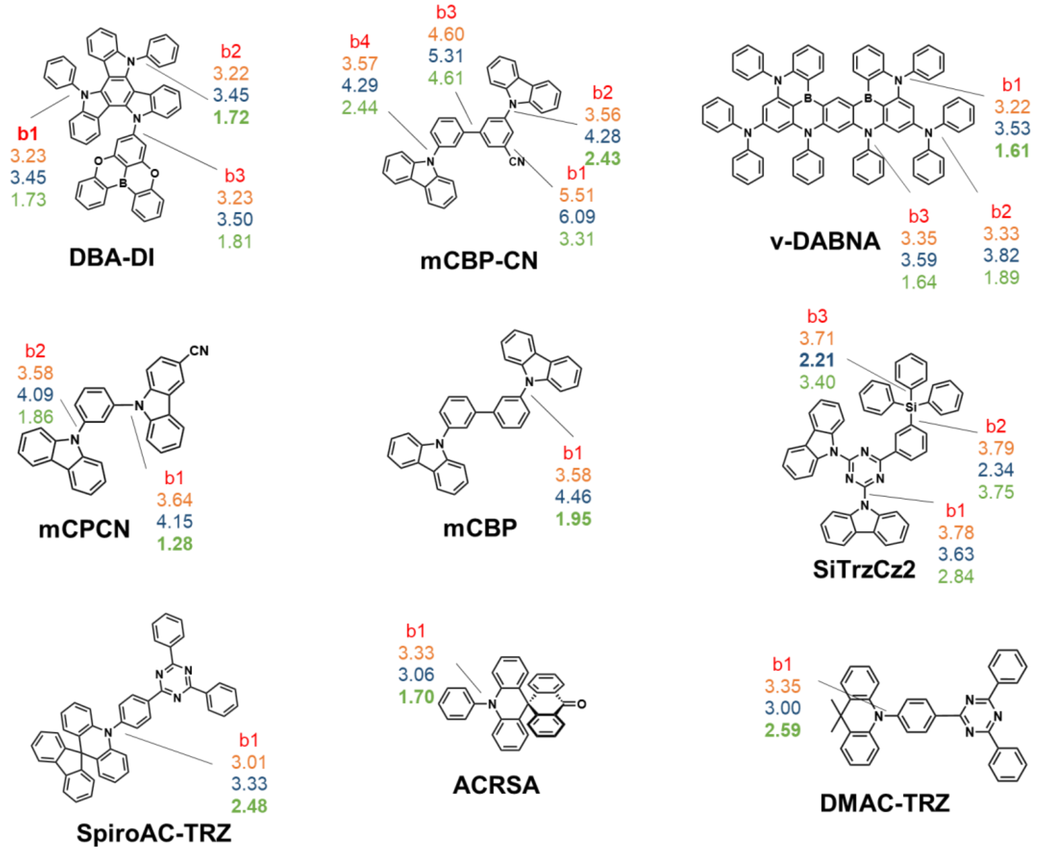

Figure S15. Bond-dissociation energies calculated for different molecules in the cationic (orange), neutral (blue), and anionic (green) state.

3. Details of Computational Simulations

Force field parametrization: All molecules were initially partitioned into rigid fragments, following the same procedure as in our previous work,[11] and all bonded parameters, apart from proper and improper dihedrals linking two rigid fragments, were taken from the OPLS-AA force field and our previous work.[11–13] The non-bonded parameters, atomic partial charges, and Lennard-Jones parameters, were then derived following the protocol, proposed by Cole *et al*,[14] by obtaining overlapping atomic electron densities via the density-derived electrostatic and chemical (DDEC6) electron density partitioning scheme.[15] The atomic partial charges can then be obtained by integrating the corresponding atomic electron densities over space. Additionally, the two Lennard-Jones parameters, A and B, are then derived using Tkatchenko−Scheffler (TS) scheme,[16] where the radius of the free atom in a vacuum is taken from reference 4.[14] The DFT electron density was obtained using Gaussian16[1] at ωB97X-D[17]/6-311+G(d,p) level and the DDEC6 computations were performed using Chargemol of version 09_26_2017.[15] After nonbonded parameters were set, the dihedral potentials that connect rigid fragments, which are usually missing in the OPLS-AA database, were then parameterized using constrained optimization scanning, performed at ωB97X- D[17]/6-311G(d,p) level using Gaussian16.[1] For more details of the parameterization of dihedral potentials, please refer to ref.[18]

MD simulations: All classical MD simulations were performed using GROMACS of version 2020.3.[19,20] A timestep of 1 fs was used across all MD simulations. For the long-range electrostatic interactions, the particle mesh Ewald (PME) method was employed with a 0.12 nm Fourier spacing. A cutoff of 13 Å was applied to all non-bonded interactions. The temperature and pressure control were accomplished using velocity rescaling with a stochastic term[21] (τ_T = 0.5 ps) and an isotropic coupling for the pressure from a Berendsen barostat (P_0 = 1 bar, $\chi = 4.5 \times 10^{-5}$ bar^{-1}, and τ_P = 0.5 ps).

For the pure DBA-DI system 2000 molecules were initially inserted at random position, but with constrained molecular rotation to ensure the alignments of molecular dipole moment, using Packmol with an initial target density of 400 kg/m^3.[22] The initial morphology of the mCBP-CN:DBA-DI host system was also generated using Packmol at a mass fraction of 60%:40% by randomly inserting 1501 mCBP-CN molecules and 551 DBA-DI molecules. As was done in the pure DBA-DI system, the molecular rotation of DBA-DI was constrained to ensure alignment of the DBA-DI molecular dipole in the initial substrate.

Both systems were then heated up from 100K to 300K at a rate of 0.67 K/ps. They were then equilibrated at 300 K until the density reached a steady value. This step helps to prevent the system from exploding due to a high heating rate. Finally, the systems were heated up from 300 K to 800 K at a rate of 0.5 K/ps, followed by an equilibration at 800 K for 10 ns. The equilibration time is long enough to ensure a steady density of the system for all compounds discussed here. Finally, the systems underwent a linear cooling procedure from 800 K to 300 K at a 500 K/ns cooling rate followed by an equilibration at 300 K for 9 ns.

DOS calculations: DoS computations were performed using the VOTCA package and used a perturbative scheme which evaluates the difference in system energies when molecules are present in their neutral or anionic form.[23,23–29]

In this work we consider electrostatic, E_{stat}, and induction energy, E_{indu}, components to the total site energy in order to evaluate a solid state correction,

$\Delta EA_s = E_{\mathrm{stat}} + E_{\mathrm{indu}}$,

to the gas phase electron affinity, EA_0. This is used to estimate the bulk electron affinity at each site,

$EA_{\mathrm{bulk}} = EA_0 + \Delta EA_s$.

The electrostatic contribution is calculated by evaluating the Coulombic sums of atom centered point charges. These point charges were calculated using distributed multipole analysis with the GDMA program and were obtained for both the neutral and anionic ground states of DBA-DI and mCBP-CN.[30]

The inductive component is evaluated using a polarizable force field, based on the Thole model, which utilizes isotropic atomic polarizabilities for each atom a on molecule i, $\alpha a, i$. The polarizable force field based off the Thole model utilizes fixed atomic polarizabilities for each element, derived from the polarizabilities of the atomic elements. These atomic polarizabilities are then rescaled to reproduce the molecular polarization volume obtained from quantum mechanics calculations at the PBE/6-311G(d,p) level,[31] and used to calculate the interaction energy between induced molecular multipoles and the permanent multipoles obtained from GDMA.[30]

The solid state EA and IE of vDABNA are approximated here by calculating the single molecule property using DFT, at the ωB97X-D[17]/6-311+G(d,p) level, in an implicit solvent with a dielectric constant corresponding to the host material, mCBP-CN. Here this was estimated to correspond to $\varepsilon_r = 3$.[1]

Frontier orbital calculations: The frontier orbitals displayed in figure four were obtained using omega tuned range-separated functionals. Here, both the ground and excited state of DBI-DA and v-DABDA were optimized using ωT-ωB97xd in combination with the Restricted Open-shell Kohn–Sham (ROKS) method.[32,33] For all the calculations, the 6-311g (d,p) basis set and the polarizable continuum model (PCM) with toluene as solvent are applied.

4. References

1. Frisch, M. J. *et al.* Gaussian 16, Version B.01. (2016).
2. Perdew, J. P., Burke, K. & Ernzerhof, M. Generalized Gradient Approximation Made Simple. *Phys. Rev. Lett.* **77**, 3865–3868 (1996).
3. Adamo, C. & Barone, V. Toward reliable density functional methods without adjustable parameters: The PBE0 model. *The Journal of Chemical Physics* **110**, 6158–6170 (1999).
4. Johnson, E. R. & Becke, A. D. A post-Hartree-Fock model of intermolecular interactions: Inclusion of higher-order corrections. *The Journal of Chemical Physics* **124**, 174104 (2006).
5. Grimme, S., Ehrlich, S. & Goerigk, L. Effect of the damping function in dispersion corrected density functional theory. *Journal of Computational Chemistry* **32**, 1456–1465 (2011).
6. Becke, A. D. & Johnson, E. R. A density-functional model of the dispersion interaction. *The Journal of Chemical Physics* **123**, 154101 (2005).

7. Grimme, S., Ehrlich, S. & Goerigk, L. Effect of the damping function in dispersion corrected density functional theory. *Journal of Computational Chemistry* **32**, 1456–1465 (2011).
8. Weigend, F. & Ahlrichs, R. Balanced basis sets of split valence, triple zeta valence and quadruple zeta valence quality for H to Rn: Design and assessment of accuracy. *Phys. Chem. Chem. Phys.* **7**, 3297–3305 (2005).
9. Ahn, D. H. *et al.* Rigid Oxygen-Bridged Boron-Based Blue Thermally Activated Delayed Fluorescence Emitter for Organic Light-Emitting Diode: Approach towards Satisfying High Efficiency and Long Lifetime Together. *Advanced Optical Materials* **8**, 2000102 (2020).
10. Effect of Substituents on the Electronic Structure and Degradation Process in Carbazole Derivatives for Blue OLED Host Materials | Chemistry of Materials. https://pubs.acs.org/doi/full/10.1021/acs.chemmater.6b02069.
11. Mondal, A. *et al.* Molecular library of OLED host materials—Evaluating the multiscale simulation workflow. *Chemical Physics Reviews* **2**, 31304 (2021).
12. Jorgensen, W. L. & Tirado-Rives, J. Potential energy functions for atomic-level simulations of water and organic and biomolecular systems. *Proceedings of the National Academy of Sciences* **102**, 6665–6670 (2005).
13. Jorgensen, W. L., Maxwell, D. S. & Tirado-Rives, J. Development and Testing of the OPLS All-Atom Force Field on Conformational Energetics and Properties of Organic Liquids. *Journal of the American Chemical Society* **118**, 11225–11236 (1996).
14. Cole, D. J., Vilseck, J. Z., Tirado-Rives, J., Payne, M. C. & Jorgensen, W. L. Biomolecular Force Field Parameterization via Atoms-in-Molecule Electron Density Partitioning. *J. Chem. Theory Comput.* **12**, 2312–2323 (2016).
15. Manz, T. A. & Limas, N. G. Introducing DDEC6 atomic population analysis: part 1. Charge partitioning theory and methodology. *RSC Advances* **6**, 47771–47801 (2016).
16. Tkatchenko, A. & Scheffler, M. Accurate Molecular Van Der Waals Interactions from Ground-State Electron Density and Free-Atom Reference Data. *Phys. Rev. Lett.* **102**, 073005 (2009).
17. Li-Ying, S. *et al.* Improving the Efficiency of Blue Organic Light-Emitting Diodes by Employing Cs-Derivatives as the n-Dopant. *Acta Physico-Chimica Sinica* **28**, 1497–1501 (2012).
18. Poelking, C. *et al.* Characterization of Charge-Carrier Transport in Semicrystalline Polymers: Electronic Couplings, Site Energies, and Charge-Carrier Dynamics in Poly(bithiophene- *alt* -thienothiophene) [PBTTT]. *The Journal of Physical Chemistry C* **117**, 1633–1640 (2013).
19. Abraham, M. J. *et al.* GROMACS: High performance molecular simulations through multi-level parallelism from laptops to supercomputers. *SoftwareX* **1–2**, 19–25 (2015).
20. Pronk, S. *et al.* GROMACS 4.5: a high-throughput and highly parallel open source molecular simulation toolkit. *Bioinformatics* **29**, 845–854 (2013).
21. Bussi, G., Donadio, D. & Parrinello, M. Canonical sampling through velocity rescaling. *The Journal of Chemical Physics* **126**, 14101 (2007).
22. Martínez, L., Andrade, R., Birgin, E. G. & Martínez, J. M. PACKMOL: A package for building initial configurations for molecular dynamics simulations. *Journal of Computational Chemistry* **30**, 2157–2164 (2009).
23. Kordt, P. & Andrienko, D. Modeling of Spatially Correlated Energetic Disorder in Organic Semiconductors. *J. Chem. Theory Comput.* 36–40 (2016).
24. Ruhle, V., Junghans, C., Lukyanov, A., Kremer, K. & Andrienko, D. Versatile object-oriented toolkit for coarse-graining applications. *Journal of Chemical Theory and Computation* **5**, 3211–3223 (2009).
25. Rühle, V. *et al.* Microscopic Simulations of Charge Transport in Disordered Organic Semiconductors. *J. Chem. Theory Comput.* **7**, 3335–3345 (2011).

26. Baumeier, B., Kirkpatrick, J. & Andrienko, D. Density-functional based determination of intermolecular charge transfer properties for large-scale morphologies. *Phys. Chem. Chem. Phys.* **12**, 11103 (2010).
27. Lukyanov, A. & Andrienko, D. Extracting nondispersive charge carrier mobilities of organic semiconductors from simulations of small systems. *Phys. Rev. B* **82**, 193202 (2010).
28. Kirkpatrick, J. An approximate method for calculating transfer integrals based on the ZINDO Hamiltonian. *Int J of Quantum Chemistry* **108**, 51–56 (2008).
29. Poelking, C. & Andrienko, D. Long-Range Embedding of Molecular Ions and Excitations in a Polarizable Molecular Environment. *J. Chem. Theory Comput.* **12**, 4516–4523 (2016).
30. Stone, A. J. Distributed Multipole Analysis: Stability for Large Basis Sets. *J. Chem. Theory Comput.* **1**, 1128–1132 (2005).
31. Perdew, J. P., Ernzerhof, M. & Burke, K. Rationale for mixing exact exchange with density functional approximations. *The Journal of Chemical Physics* **105**, 9982–9985 (1996).
32. Stein, T., Eisenberg, H., Kronik, L. & Baer, R. Fundamental Gaps in Finite Systems from Eigenvalues of a Generalized Kohn-Sham Method. *Phys. Rev. Lett.* **105**, 266802 (2010).
33. Kronik, L., Stein, T., Refaely-Abramson, S. & Baer, R. Excitation Gaps of Finite-Sized Systems from Optimally Tuned Range-Separated Hybrid Functionals. *J. Chem. Theory Comput.* **8**, 1515–1531 (2012).
